# Released micromachined beams utilizing laterally uniform porosity porous silicon

**DOI:** 10.1186/1556-276X-9-426

**Published:** 2014-08-22

**Authors:** Xiao Sun, Adrian Keating, Giacinta Parish

**Affiliations:** 1 School of Mechanical and Chemical Engineering, University of Western Australia, 35 Stirling Hwy, Crawley, Western Australia 6009, Australia; 2 School of Electrical, Electronic and Computer Engineering, University of Western Australia, 35 Stirling Hwy, Crawley, Western Australia 6009, Australia

**Keywords:** Porous silicon, Photolithography, Microbeams, Release

## Abstract

Suspended micromachined porous silicon beams with laterally uniform porosity are reported, which have been fabricated using standard photolithography processes designed for compatibility with complementary metal-oxide-semiconductor (CMOS) processes. Anodization, annealing, reactive ion etching, repeated photolithography, lift off and electropolishing processes were used to release patterned porous silicon microbeams on a Si substrate. This is the first time that micromachined, suspended PS microbeams have been demonstrated with laterally uniform porosity, well-defined anchors and flat surfaces.

**PACS:**

81.16.-c; 81.16.Nd; 81.16.Rf

## Background

Porous silicon (PS), which is normally formed via the partial electrochemical dissolution of crystalline silicon in a HF/ethanol solution [1], has gained significant attention due to its biocompatibility and stability. With a large surface area and easily tunable porosity (which directly determines the refractive index), PS has been demonstrated in applications including light emitting diodes [2], sensors [3,4] and photo detectors [4,5]. However, previously reported PS tunable microelectromechanical system (MEMS) devices for gas sensors [6], biological sensors [7] and optical filters [8,9] have mainly been fabricated through a predefined patterning process utilizing a defined pattern or mask on Si prior to anodization, resulting in unwanted under-mask etching and very low lateral uniformity in PS films. The predefined patterning technique limits complementary metal-oxide-semiconductor (CMOS) compatibility of the process for making further complex structures [6], limiting PS use as a separate material in MEMS device fabrication.

PS-suspended structures can provide increased sensitivity in MEMS devices through the large surface area and the ability to use porosity to control mechanical properties [10-12]. Sensing using released microbeams has been studied for a variety of materials, including Si, Si_3_N_4_ and AlN [13-15]. Suspended PS structures have previously been fabricated and released [12,16], but the porosity of those films was not uniform, leading to significant bending from internal stress, made worse by the very low stiffness of the material. Furthermore, previous PS MEMS have been large or poorly defined [7,8]. This negates a significant advantage of MEMS, which is that their small size provides both robustness against inertial effects and high resonance, the latter being essential for high sensitivity biosensors [17]. Uniform porosity and well-defined porous silicon patterning is required to achieve a high-quality MEMS technology. Furthermore the process must be compatible with a high-volume (scalable) manufacture process. Lai *et al.* demonstrated a process based on N_2_ annealing which reduced oxidation in ambient air and made the films compatible with standard CMOS photolithography [18]. This approach makes PS a suitable platform for creating patterned structures of uniform porosity, and allows multistep processing through repeated anodization, annealing and photolithography to be performed.

In this work, we demonstrate that well-defined, laterally uniform porosity PS microbeams can be successfully fabricated and released. A process based on anodization, annealing, RIE, repeated photolithography, lift off and electropolishing is presented, which is designed with CMOS compatibility in mind. Process yield along with length of microbeam was studied, and surface profilometry of fabricated structures of PS microbeams was performed. The surface profile shows that this approach yields PS microbeam with small surface variation, showing well-defined PS structures were fabricated.

## Methods

The wafer material used was moderately doped p-type (100) silicon with resistivity of 0.08 to 0.10 Ω · cm. Room temperature anodization was performed in a 15% HF/ethanol solution, unless otherwise specified. PS films in this paper were anodized using a current density of 10 mA/cm^2^ for 403 s and subsequently annealed in N_2_ atmosphere at 600°C for 6 min, to create low-temperature annealed porous silicon films with porosity *P* = 81% and a physical thickness of *t* = 2.45 μm. The annealing process is critical as it makes the PS film suitable for direct photolithography processing using alkaline developers [18]. This type of PS was used in the work reported here, as its characterization and annealing has been previously comprehensively studied [19,20]. However, as part of the investigations, it was confirmed that PS films with different porosity and thickness are also suitable. The PS microbeams under investigation here were designed and fabricated with dimensions *L* × *W* × 2.45 μm, where 80 μm < *L* < 1,000 μm and 20 μm < *W* < 50 μm.

**Figure 1:**
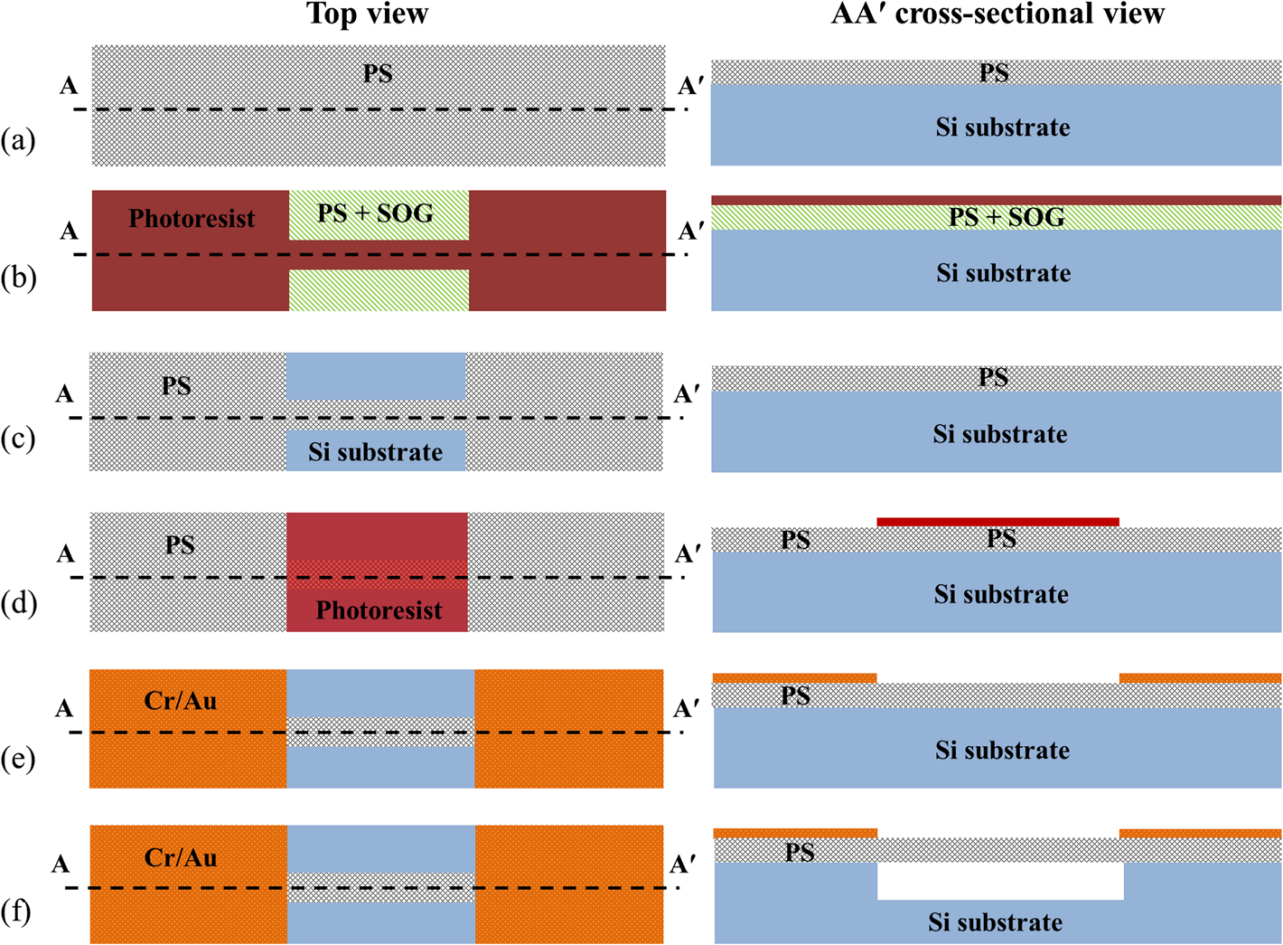
**Process to achieve released PS microbeams. (a)** After PS formation and N_2_ annealing, **(b)** after first photolithographic step, **(c)** after RIE of PS and then removal of photoresist, **(d)** after second photolithographic step, **(e)** after metal lift-off and **(f)** after electropolishing and critical point drying.

The PS beams were machined using standard CMOS processes of repeated photolithography using positive and negative resists, lift-off and plasma etching [21,22]. Figure 1 shows the structure at various stages of the PS microbeam fabrication process. First, an anodized PS film was created and subsequently annealed under conditions described above, as shown in Figure 1a. Then, a layer of spin-on glass (SOG) was spun on the annealed PS film prior to the application of the photoresist layer, to fill the pores, preventing photoresist seepage into PS. The SOG (700B, 10.8% SiO_2_ content, Filmtronics Inc., Butler, PA, USA) was spun twice at a speed of 2,000 rpm for 40s each time. Microbeams and anchors were defined using a standard positive photoresist photolithographic process using AZ EBR solvent (MicroChemicals GmbH, Ulm, Germany) diluted positive photoresist AZ6632 (MicroChemicals, 20% solid content, 0.85-μm thick), as shown in Figure 1b. After photolithographic patterning, the SOG everywhere in the PS was removed by a short 10-s dip in 10% HF/ethanol, which resulted in an as-fabricated PS film selectively covered by photoresist. Inductively coupled plasma reactive ion etching (ICP-RIE) was used to rapidly etch (1 μm/min for the as-fabricated PS in this work [23]) the PS film in the region not covered by photoresist to form the PS beam and anchor regions. ICP-RIE was done with a gas mixture of CF_4_/CH_4_ (31 sccm/3 sccm) at a temperature of 25°C. If the SOG in the uncovered PS has not been totally removed, the RIE rate will decrease dramatically, which results in a much longer etching time to remove the PS film, providing a process indicator of thorough SOG removal from the pores. After etching, the positive photoresist was removed in acetone, leaving the patterned PS consisting of microbeams and anchors, as shown in Figure 1c.

After that, a second standard photolithographic process using negative photoresist AZ2070 (MicroChemicals, 6.8-μm thick) was employed to define a metal mask pattern up to the anchor, as shown in Figure 1d. A Cr/Au (10/200 nm) layer was subsequently deposited on to anchor regions with a lift-off process based on the second photolithography, as shown in Figure 1e. The negative photoresist was removed by a 15-min *N*-methyl-2-pyrrolidone (NMP) or dimethyl sulfoxide (DMSO) dip and a 5-min acetone dip in the lift-off process. The metal region over the PS was important to define the anchors during electropolishing as described later. Electropolishing with HF-based electrolyte was carried out to etch the Si, and the electrolyte ensured any residual SOG in the pores was removed. Electropolishing was carried out using a similar process to anodization, but with different electrolyte (a 3% HF/DI solution) and electrical conditions (20 mA/cm^2^, 180 s). After electroplishing, PS microbeams suspended on top of Si substrate were formed which were kept submerged until release. The samples were rinsed in DI water wash and transferal to a methanol bath during the critical point drying process used to release the PS doubly clamped microbeams illustrated in Figure 1f.

## Results and discussion

**Figure 2:**
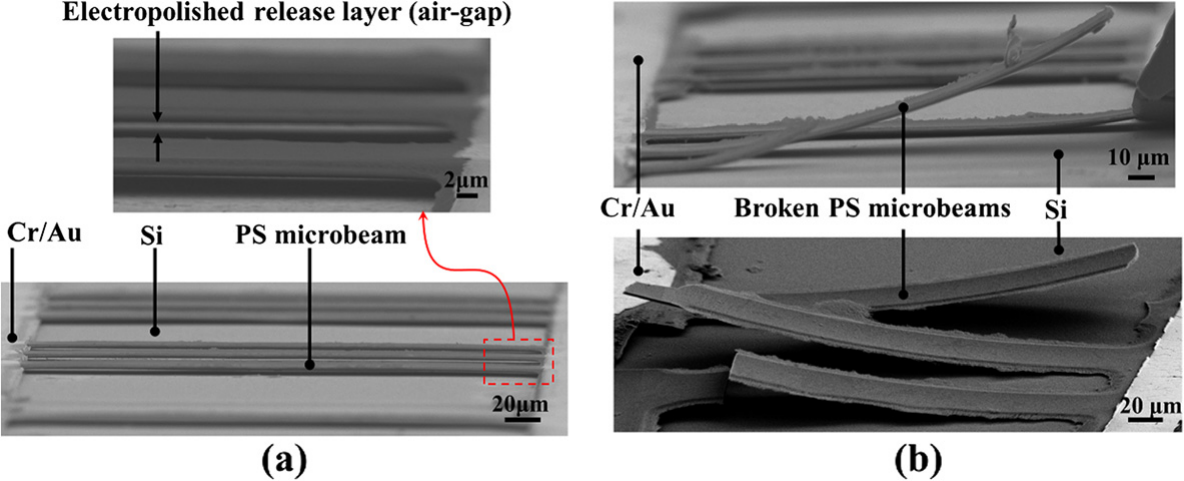
**SEM images of released PS microbeams.** Beam voltage of 5 kV. **(a)** Released doubly clamped microbeams; the length of the microbeams was 300 μm and the width was 25 μm; **(b)** broken PS microbeams which formed single end fixed beams.

Using the above processes, a complete fabrication procedure to successfully release high-porosity meso-porous microbeams was achieved for the first time. Figure 2a,b shows SEM micrographs of the released microbeams and anchors. As shown in Figure 2a, 300-μm-long doubly clamped microbeams (microbridges) were well defined and suspended approximately 2 μm above the Si substrate, where the gap was as defined by the electropolishing duration. Figure 2b shows broken microbeams after fabrication, resulting in microbeams suspended above Si which were fixed only on one end. The upwardly bent profile of the microbeams indicated that stress gradient in the PS film, most likely due to porosity gradients and the metal layer [24,25], are significant; however, cantilever studies of stress gradient are outside of the scope of this work.

**Figure 3:**
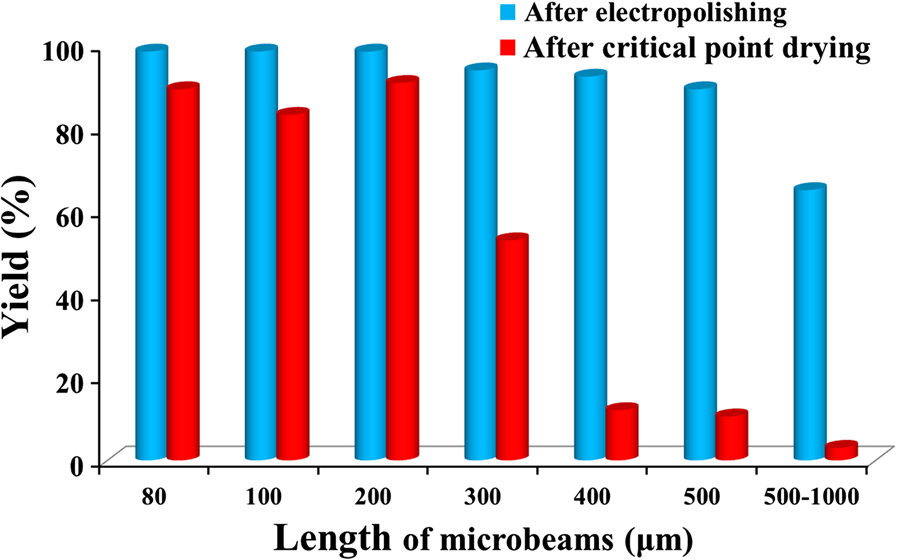
Yields of doubly clamped microbeams after electropolishing and after critical point drying.

Figure 3 shows the measured yields of 66 doubly clamped microbeams after electropolishing and critical point drying as a function of microbeam length. As demonstrated from the data, yields of the microbeams were high after electropolishing. However, after the critical point drying, the yield was only high (>50%) for microbeams shorter than 300 μm, dropping significantly for microbeams above 300 μm in length. Although critical point drying is expected to achieve better results than other drying approaches [26,27], the rigidity of the beams drops as *L*^4^ under uniform loading [28], which combined with the very low Young’s modulus of PS (near that of rubber), compromises the integrity of microbeams much longer than 300 μm during the drying process. The factors that impact rigidity of PS microbeams including internal stress and stress gradient are still under investigation to understand and improve the yield.

**Figure 4:**
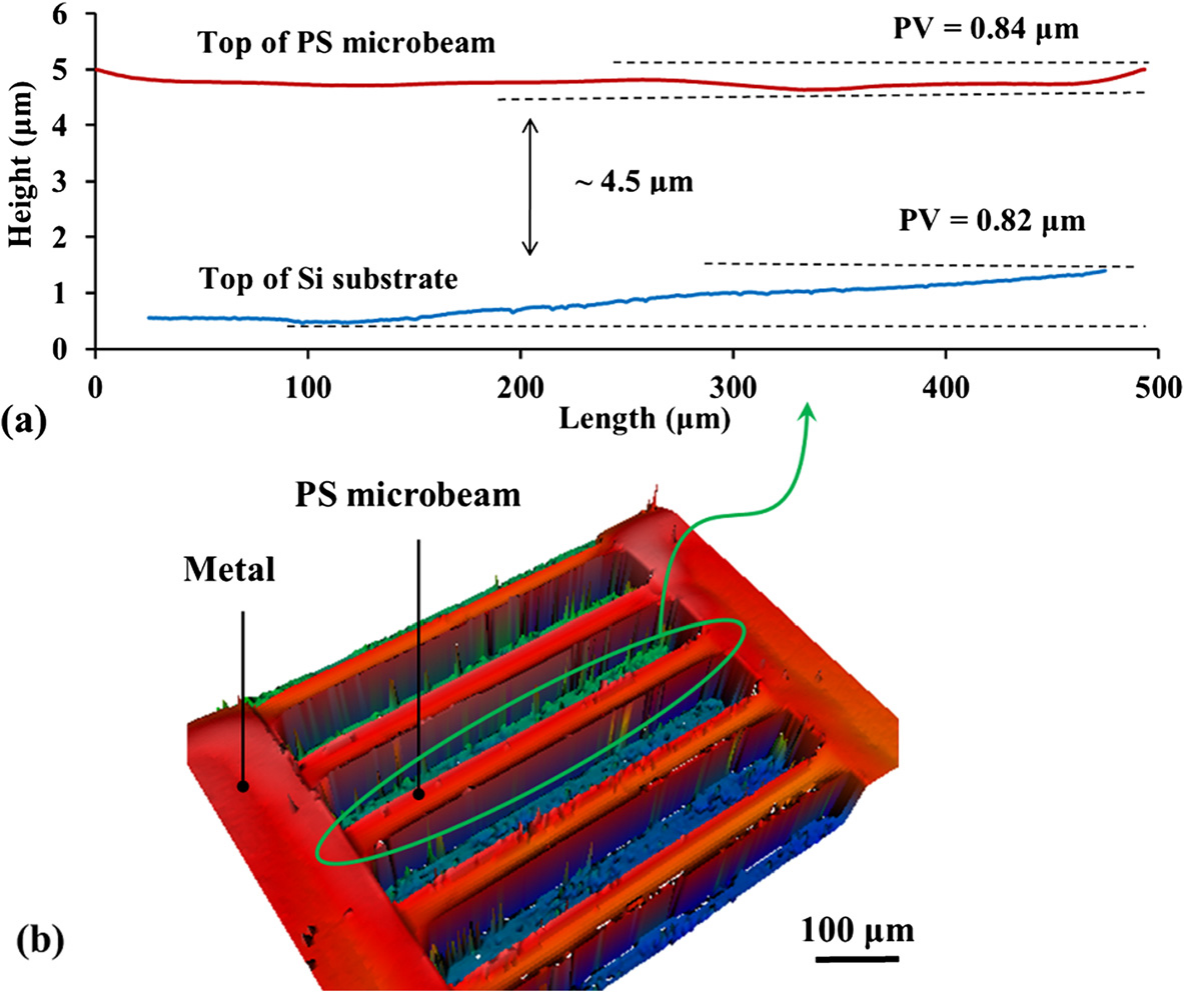
**Surface profile of released doubly clamped microbeam. (a)** Plot of PS doubly clamped microbeam and Si substrate, **(b)** 3D plot of PS doubly clamped microbeam. The length of microbeam was 500 μm and the width was 25 μm.

The profile of one of the longest released PS microbeams measured using an optical profilometer is shown in Figure 4. The microbeams were 500 μm in length and 25-μm wide. Electropolishing resulted in the doubly clamped microbeam being suspended 2 μm above the Si substrate, giving a total distance from substrate to the PS top surface of 4.5 μm. For this beam the peak-to-valley (PV) variation in the surface topology was 0.84 μm, while the substrate PV variation after electropolishing was 0.82 μm. The PS surface deformation is attributed to compressive stress in the released film as it is well known that as-fabricated PS is compressively stressed due to the presence of dihydride [29] which increases the lattice spacing.

**Figure 5:**
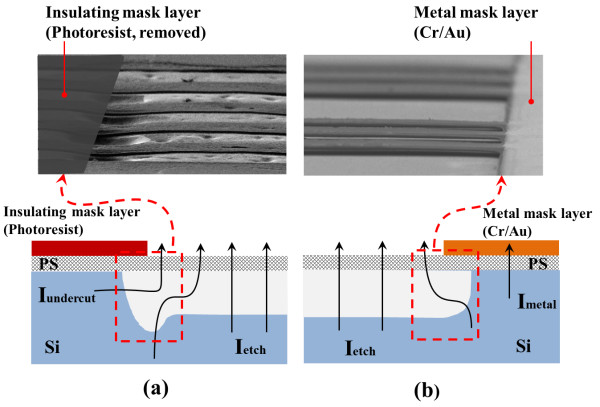
**Comparison of undercut profiles resulting from electropolishing. (a)** Insulating mask layer (photoresist), **(b)** conductive mask layer (metal).

The masking material during the electropolishing step was investigated to optimize the release process. While the RIE defined the PS beam and anchor regions, it was the masking layer used during electropolishing that defined the anchor itself. It was found that use of a metal layer to define the anchor of the microbeams was critical to control the electric field during electropolishing. Figure 5 shows a comparison of released microbeams and a schematic illustration of the undercut profiles, resulting from electropolishing with an insulating mask layer (photoresist) and a conductive masking layer (metal). Significant and non-uniform undercutting occurred when using an insulating mask layer, compared with minimal undercut from the metal masking layer. This was consistent with previous reports that the use of an insulating mask such as photoresist rather than metal resulted in a large undercut [30].

**Figure 6:**
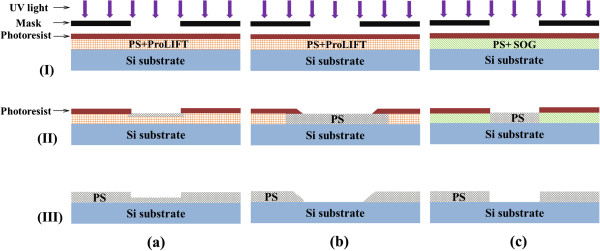
**Comparison of pore fill techniques utilizing ProLIFT and SOG.** Different techniques: **(a)** ProLIFT pore filling technique with short developing time, **(b)** ProLIFT pore filling technique with long developing time and **(c)** SOG pore filling technique. At three steps: (I) UV light exposure with photoresist patterning, (II) developing to remove exposed positive photoresist and (III) RIE and photoresist/pore filling material removal.

During the fabrication process, SOG was employed to fill the PS pores in place of a polymer (ProLIFT) used in our previous work [31]. To understand the improvement from using SOG, a comparison of pore filling techniques utilizing ProLIFT and SOG is depicted schematically in Figure 6, at three photolithography steps: (I) UV light exposure with photoresist patterning, (II) developing to remove exposed positive photoresist and (III) RIE and photoresist/pore filling material removal. While ProLIFT can be used to fill the PS pores prior to the application of photoresist in step I, it is not UV sensitive but can be removed by standard alkaline developer during the photoresist development step. This allows ProLIFT to be patterned in the same wet process that defines the photoresist but requires accurate timing of the development time. If the developing time is too short, exposed photoresist will be removed but ProLIFT residue will remain in the PS film slowing the RIE removal of PS, as shown in Figure 6a. Furthermore, any residual ProLIFT in the PS film once released is expected to introduce stress in released microbeams, resulting in beam breakage (poor yield). On the other hand, if the developing time is too long, the photoresist will be over developed, causing a large side wall angle of the photoresist pattern, resulting in poorly defined PS structures as shown in Figure 6b. Worse, over developing can result in lift off of the patterned photoresist if it is not well attached to the PS film. Repeated experiments have shown the development time when using ProLIFT becomes a significant issue when patterning PS films above 1-μm thick, as they require a much longer developing time (>60 s) to remove all the ProLIFT in the PS films than typically required for photoresist development (approximately 30 s).

On the contrary, SOG can be used to form a layer of SiO_2_ to fill the pores of PS at step I of Figure 6, which is not removed during the developing process at step II. This guarantees the accurate control of developing time for the photoresist layer, resulting in well-patterned PS structures at step III, as shown in Figure 6c. Our tests showed a 10-s dip in 10% HF/DI is sufficient to remove all SOG in an exposed PS film (where there was no photoresist) up to 2.45-μm thick. The short dip resulted in an optical thickness change of less than 4.4%, suggesting the short dip had very little effect on the PS layer. In this work which used PS layers of 2.45-μm thickness**,** SOG as a pore filling layer was more advantageous than ProLIFT and was used as described.

These results show a complete MEMS fabrication process using a single material system can be achieved using combination of anodization and electropolishing. No sacrificial layer was required to achieve release of the beams. This is fundamentally different from traditional MEMS processing and has the potential to resolve interface compatibility issues such as differences in thermal coefficient of expansion. The thickness of the PS beam (2.45 μm) and porosity (81%) were chosen to achieve the same rigidity as an a-Si beam of thickness 0.6 μm. This allowed us to demonstrate the fabrication process on extremely high-porosity meso-porous silicon, which is well suited to sensing applications due to its very large surface area [3,32]. The high porosity and high thickness balance to produce an expected resonant frequency in the range of 16 to 400 kHz for microbeams with length of 100 to 500 μm. Variation of porosity and thickness are also options to adjust frequency of beams (not detailed in this work). Residual and stress gradients in the films need to be studied to allow both doubly clamped and cantilever structures to be fabricated, as these are the basis on most MEMS devices. We are aware that the use of Au as part of the metallisation scheme would prevent implementation in some CMOS foundries. Our investigations have been limited to metals currently available in our facility; however, alternative metallisation or doping could be used to replace the Cr/Au layers for the electropolishing steps to achieve a completely CMOS-compatible process.

## Conclusions

This work has demonstrated micromachined, suspended PS microbeams with laterally uniform porosity and structurally well-defined beams. We have demonstrated repeated photolithographic processing on PS films that is compatible with CMOS processes; however, for complete CMOS integration, a different metallisation may be required to avoid use of Cr/Au. A deposited metal mask layer was used during electropolishing to ensure a uniform electric field and minimal underetching of the PS layer. A new pore filling technique using SOG allowed the use of thick (2.45 μm) films. The surface profile of the released microbeams indicated well-defined structures. This approach demonstrates a method of fabricating complex PS structures using a scalable PS-MEMS technology.

## Abbreviations

PS: porous silicon; CMOS: complementary metal-oxide-semiconductor; MEMS: microelectromechanical system; SOG: spin on glass; rpm: revolutions per minute; ICP-RIE: inductively coupled plasma reactive ion etcher; NMP: *N*-methyl-2-pyrrolidone; DMSO: dimethyl sulfoxide; RMS: root mean square.

## Competing interests

The authors declare that they have no competing interests.

## Authors’ contributions

XS carried out the experiments, undertook fabrication steps, measured the microbeams, contributed to the interpretation of the data and drafted the manuscript. AK contributed to the guidance of the fabrication process, measurement of microbeams, interpretation of the data and drafting of the manuscript. GP contributed to the guidance and input to fabrication process and manuscript. All authors read and approved the final manuscript.

## Authors’ information

XS received the B.Sc. degree and the M.Sc. degree in optics from Xi’an Jiaotong University, Xi'an, China, in 2005 and 2008. In 2008, he joined the State Intellectual Property Office of China, working on extensive examination of patent applications in the areas of measuring devices and microelectromechanical systems. Since 2012, he has been working toward the Ph.D. degree in microelectronic engineering at The University of Western Australia, Perth, Australia. His thesis focuses on micromachining applications based on porous silicon. GP received the B.S. degree in Chemistry in 1995 and the bachelors and M.Sc. degrees in Electronic Engineering in 1995 and 1997, respectively, all from The University of Western Australia, Perth, and the Ph.D. degree in Electrical Engineering in 2001, from the University of California, Santa Barbara. She joined The University of Western Australia as an Australian Postdoctoral Fellow in 2001 and is now a professor at the same institution. Her main research interests are III-V nitride and porous silicon materials and devices. Specific interests within these areas currently include development of processing technology, transport studies and development of novel chem- and bio-sensors. AK received the bachelors and Ph.D. degrees in Electrical/Electronic Engineering in 1990 and 1995, respectively, from the University of Melbourne. He worked as a post-doctoral fellow at NTT (Musashinoshi, Japan) from 1996 and joined the UC Santa Barbara (USA) in 1998. He joined Calient Networks, Santa Barbara in 1999 as the Fiber Optics Technology Manager. In 2004, he joined the University of Western Australia as a research fellow and became an assistant professor in 2007 and a professor in 2010. He received the DSTO Eureka Prize for Outstanding Science in Support of Defence or National Security in 2008 for his contributions to the development of a MEMS microspectometer, and his current research interests include porous silicon for micromachined devices, optical MEMS biosensors, and microfluidics.
